# Supplementary material for: Cortical ChAT+ neurons co-transmit acetylcholine and GABA in a target- and brain-region-specific manner
Source: eLife. 2020 Jul 2;9:e57749. doi: 10.7554/eLife.57749 (PMC7360370; doi:10.7554/eLife.57749)
Supplement: Transparent reporting form [file elife-57749-transrepform.docx]

***eLife’s* transparent reporting form**

We encourage authors to provide detailed information *within their submission* to facilitate the interpretation and replication of experiments. Authors can upload supporting documentation to indicate the use of appropriate reporting guidelines for health-related research (see [EQUATOR Network](http://www.equator-network.org/%20)), life science research (see the [BioSharing Information Resource](https://biosharing.org/)), or the [ARRIVE guidelines](http://www.plosbiology.org/article/info:doi/10.1371/journal.pbio.1000412) for reporting work involving animal research. Where applicable, authors should refer to any relevant reporting standards documents in this form.

If you have any questions, please consult our Journal Policies and/or contact us: [editorial@elifesciences.org](mailto:editorial@elifesciences.org).

**Sample-size estimation**

- You should state whether an appropriate sample size was computed when the study was being designed
- You should state the statistical method of sample size computation and any required assumptions
- If no explicit power analysis was used, you should describe how you decided what sample (replicate) size (number) to use

Please outline where this information can be found within the submission (e.g., sections or figure legends), or explain why this information doesn’t apply to your submission:

- In general, experiments in this study are descriptive and not comparative, so we did not perform sample-size estimation.
- For connectivity surveys, we recorded from a minimum of 30 cells per condition. Assuming a low rate of connectivity of 10%, the probability of seeing no responses is p = 0.042 (Binomial distribution with probability of 0.1, 0 responses for 30 trials).
- For comparative analysis in Figure 6A-D, we made a post-hoc comparison after gathering data in two brain regions and noticing an unexpected difference, which was significant by a Fisher’s exact test. We therefore did not perform sample-size estimation for these experiments ahead of time.

**Replicates**

- You should report how often each experiment was performed
- You should include a definition of biological versus technical replication
- The data obtained should be provided and sufficient information should be provided to indicate the number of independent biological and/or technical replicates
- If you encountered any outliers, you should describe how these were handled
- Criteria for exclusion/inclusion of data should be clearly stated
- High-throughput sequence data should be uploaded before submission, with a private link for reviewers provided (these are available from both GEO and ArrayExpress)

Please outline where this information can be found within the submission (e.g., sections or figure legends), or explain why this information doesn’t apply to your submission:

For all immunohistochemistry, fluorescent *in situ* hybridization data, and electrophysiology data (Figures 1-3,6,7), each individual cell is considered a biological replicate. The number of cells for each condition are indicated in the figure legend, in the figure itself, or both.

For array tomography (Figure 4), an image stack containing many individual cells and fluorescently labeled pre-synaptic terminals are considered a biological replicate. The number of individual image stacks, taken from different mice, are indicated in the figure legends.

For labeling of individual pre-synaptic (Figure 5), we consider an image stack taken from each mouse as a biological replicate. However, we then considered each pre-synaptic terminal separately and pooled pre-synaptic terminals across replicates. Both the number of mice that samples were taken from and the number of pre-synaptic terminals analyzed are indicated in the figure legend.

**Statistical reporting**

- Statistical analysis methods should be described and justified
- Raw data should be presented in figures whenever informative to do so (typically when N per group is less than 10)
- For each experiment, you should identify the statistical tests used, exact values of N, definitions of center, methods of multiple test correction, and dispersion and precision measures (e.g., mean, median, SD, SEM, confidence intervals; and, for the major substantive results, a measure of effect size (e.g., Pearson's r, Cohen's d)
- Report exact p-values wherever possible alongside the summary statistics and 95% confidence intervals. These should be reported for all key questions and not only when the p-value is less than 0.05.

Please outline where this information can be found within the submission (e.g., sections or figure legends), or explain why this information doesn’t apply to your submission:

The statistical tests used and exact p-values we report are present in the figure legends (Figure 6) or in the main text. We did not make multiple comparisons. Median values are graphed in addition to individual data points, with SEM, as indicated in the figure legends.

Where possible, we graphed or indicated the classification each cell or pre-synaptic terminals we analyzed (Figures 3,5,6,7).

(For large datasets, or papers with a very large number of statistical tests, you may upload a single table file with tests, Ns, etc., with reference to sections in the manuscript.)

**Group allocation**

- Indicate how samples were allocated into experimental groups (in the case of clinical studies, please specify allocation to treatment method); if randomization was used, please also state if restricted randomization was applied
- Indicate if masking was used during group allocation, data collection and/or data analysis

Please outline where this information can be found within the submission (e.g., sections or figure legends), or explain why this information doesn’t apply to your submission:

This study did not use group allocation. All mice used were 2-4 months of age, and the experimental condition was known to the experimenter for all connectivity experiments (Figures 3,5,6). For all imaging data sets, wild-type, ChAT-ires-Cre, or VIP-ires-Cre mice were used, and analysis was automated to prevent experimenter bias. This information can be found in the methods section.

**Additional data files (“source data”)**

- We encourage you to upload relevant additional data files, such as numerical data that are represented as a graph in a figure, or as a summary table
- Where provided, these should be in the most useful format, and they can be uploaded as “Source data” files linked to a main figure or table
- Include model definition files including the full list of parameters used
- Include code used for data analysis (e.g., R, MatLab)
- Avoid stating that data files are “available upon request”

Please indicate the figures or tables for which source data files have been provided:

Source data has been made available for all figures at: https://dataverse.harvard.edu/dataset.xhtml?persistentId=doi:10.7910/DVN/AIUTNJ
